# Learning Basketball Tactical Actions from Video Modeling and Static Pictures: When Gender Matters

**DOI:** 10.3390/children8111060

**Published:** 2021-11-17

**Authors:** Ghazi Rekik, Yosra Belkhir, Nourhen Mezghanni, Mohamed Jarraya, Yung-Sheng Chen, Cheng-Deng Kuo

**Affiliations:** 1 Research Laboratory: Education, Motricity, Sport and Health (LR19JS01), High Institute of Sport and Physical Education, Sfax University, Sfax 3000, Tunisia; ghazi.rek@gmail.com (G.R.); jarrayam@yahoo.fr (M.J.); 2 Tanyu Research Laboratory, Taipei 112, Taiwan; 3 Department of Physical Education, Al-Udhailiyah Primary School for Girls, Al-Farwaniyah 085700, Kuwait; belkhir.ysr@gmail.com; 4 High Institute of Sport and Physical Education, Manouba University, Manouba 2010, Tunisia; 5 Department of Physical Education and Sport Science, Taif University, Taif 26571, Saudi Arabia; nsmezghanni@tu.edu.sa; 6 High Institute of Sport and Physical Education, Sfax University, Sfax 3000, Tunisia; 7 Department of Exercise and Health Sciences, University of Taipei, Taipei 111, Taiwan; 8 Exercise and Health Promotion Association, New Taipei City 241, Taiwan; 9 Department of Medical Research, Taipei Veterans General Hospital, Taipei 112, Taiwan; 10 Department of Medicine, Taian Hospital, Taipei 104, Taiwan

**Keywords:** video modeling, static pictures, motor learning, gender difference, basketball, physical education

## Abstract

Recent studies within the physical education domain have shown the superiority of dynamic visualizations over their static counterparts in learning different motor skills. However, the gender difference in learning from these two visual presentations has not yet been elucidated. Thus, this study aimed to explore the gender difference in learning basketball tactical actions from video modeling and static pictures. Eighty secondary school students (M_age_ = 15.28, SD = 0.49) were quasi-randomly (i.e., matched for gender) assigned to a dynamic condition (20 males, 20 females) and a static condition (20 males, 20 females). Immediately after watching either a static or dynamic presentation of the playing system (*learning phase*), participants were asked to rate their mental effort invested in learning, perform a game performance test, and complete the card rotations test (*test phase*). The results indicated that spatial ability (evaluated via the card rotations test) was higher in males than in female students (*p* < 0.0005). Additionally, an interaction of gender and type of visualization were identified, supporting the ability-as-compensator hypothesis: female students benefited particularly from video modeling (*p* < 0.0005, *ES* = 3.12), while male students did not (*p* > 0.05, *ES* = 0.36). These findings suggested that a consideration of a learner’s gender is crucial to further boost learning of basketball tactical actions from dynamic and static visualizations.

## 1. Introduction

In recent years, technology has been gaining increasing importance in different learning environments. Within the physical education (PE) domain, technology has also become an integral part of the curriculum and instruction [1]. In fact, while highly advanced and sophisticated forms of technology are generally not available during PE lessons [2], dynamic visualizations such as video modeling examples remain the more readily available didactical tools for teachers to present and explain various motor skills [3]. Video modeling involves showing the student a recording of the expert performance of a motor skill [4,5]. According to Hoogerheide et al. [6], video modeling examples seem to be very well suited for motor learning, due to their capability to deliver the information concerning how to perform a skill in an accurate way. Inspired by Bandura’s social learning theory [7], a consistent body of research carried out in the PE setting has shown the effectiveness of observational learning through model-based videos. For example, it was found that video modeling examples enable inexperienced climbers to perceive and accomplish new possibilities for action and facilitate their climbing performances [8]. Moreover, a model-based video has been observed to be better than a videotaped replay of one’s own performance for the acquisition and retention of the set and serve skills in volleyball [5]. Furthermore, viewing a skilled model was more effective than oral explanations for the acquisition of the shooting skill in handball [9]. In the same vein, Barzouka et al. [10] showed that observing a skilled model performing the skill of a pass in volleyball was more beneficial than oral explanations. According to Carroll and Bandura [11], observing a model would provide a perceptual blueprint of symbolic codes of the observed action. These blueprints will guide subsequent performance. In addition, Pollock and Lee [12] explained that modeling is an effective teaching method because actions which are tricky to describe verbally often can be demonstrated visually.

Despite these claims, based on the cognitive load theory [13] (a theory that considers how visual or auditory information impacts working memory and learning), Wong et al. [14] argued that videos modeling examples may not always be effective for learning, as they can become subject to transience effects. The transient information effect can be observed with videos or animations that provide a non-permanent flow of information that disappears from the computer screen [13]. Transient information requires that learners have to maintain previously presented information in WM in order to integrate it with later information [15,16]. These mental activities can overload the memory system and cause an overflow of the WM capacity [17]. The negative transient information effect provides a possible explanation as to why empirical research in the scope of cognitive load theory has suggested that replacing videos with a set of permanent/static pictures, describing the essential states of the dynamic event, may reduce the extraneous cognitive load. This instructional strategy allows learners to benefit from sufficient time to identify and process relevant information and effectively integrate it in long-term memory [18]. Moreover, viewing a series of static pictures offers the possibility to revise and compare different parts of the display as frequently as desired [19].

In this context, examining the effectiveness of video modeling vs. static pictures in learning different motor skills has raised considerable interest among sport didacticians/psychologists [20]. These scientific works dovetail nicely with findings from observational learning research. For example, Rekik et al. [21] showed that viewing skilled models performing tactical actions in basketball was more effective than viewing a series of simultaneous static pictures in terms of cognitive load, game comprehension, and attitudes (e.g., attention and enjoyment). Additionally, Rekik and his colleagues [22] recommended, whatever the complexity of the playing system, the use of video modeling examples (rather than static pictures) to teach and/or learn tactical actions in basketball. More recently, it was established that observing a skilled model performing a judo technique (through video) generated better recall-performances and guaranteed better motivation levels than different presentations of static pictures in university PE students [23,24]. The activation of the mirror neuron system has been particularly adopted by these scientists to argue the superiority of model-based videos over static visualizations (i.e., a series of photographs) in learning sport-motor knowledge/skills. This system was originally identified in primates, representing a neurophysiological circuit distributed across the pre-motor cortex that is automatically activated when someone is observing another person performing an action [25,26]. Additionally, as humans’ actions are part of primary knowledge, their acquisition is very easy and requires little cognitive effort [27]. Consequently, viewing dynamic visual tools involving motor skills does not require excessive cognitive resources, because humans are biologically evolved to effectively acquire such kinds of knowledge. The phenomenon of learning human actions through video modeling examples is referred to as “the human movement effect” [27].

The major limitation of the above-mentioned studies examining the relative effectiveness of video modeling versus static pictures in learning is that the gender of learners has not been taken into consideration. Indeed, the gender difference in learning from dynamic and static visualizations has been reported in previous educational research carried out in non-sporting domains, yielding to discrepant results [28]. On the one hand, some studies have shown that animations were especially helpful for males in geographic and problem-solving learning, indicating that while males outperformed females with the animated presentation, both genders performed similarly under static and animated presentations [29,30]. This group of scientific works supports the “ability-as-enhancer hypothesis”, indicating that high spatial ability learners benefited particularly from dynamic visualizations, and low spatial ability learners did not [31,32]. Indeed, it well established that spatial ability is higher in males than females [33,34,35,36,37]. On the other hand, another group of studies have shown that instructional animations were particularly helpful for females in learning chemistry and physical science topics [38,39]. Similarly, it was found that there is a significant presentation–gender interaction when learning a manipulative motor skill (i.e., Lego construction task), indicating that while female students outperformed males at the completion test with video modeling, no gender differences were found with the static presentation [40]. This second group of scientific works supports the “*the ability-as-compensator hypothesis*”, indicating that low spatial ability learners (i.e., females) profited mainly from dynamic visualizations, and high spatial ability learners (i.e., males) did not [31,32,41].

While the gender difference in learning from dynamic and static visualizations was explored across a broad range of instructional domains, no explicit investigation has been conducted to examine the relationships between these two visual representations and learners’ gender in learning motor skills in the PE/sport domain (i.e., motor skills requiring the whole body). We attempted to fill this knowledge gap via the present experiment, by exploring the gender difference in learning basketball tactical actions from video modeling and simultaneous static pictures in secondary school students. It was hypothesized that video modeling would be more beneficial than static pictures for learning tactical actions in basketball. It was also hypothesized that there would be a gender–instructional visualization interaction (it was an open question as to which gender would benefit most in this study due to the mixed results of previous non-sporting studies).

## 2. Materials and Methods

### 2.1. Participants

Eighty students (M_age_ = 15.28, SD = 0.49; 50% females) from a public secondary school in Tunisia completed the experimental procedure of the current study. They were selected based on sample convenience and school administrative support. The required sample size was calculated as 80 with alpha level of 0.05, the power of 0.80, and the effect size of 0.46 derived from a previous study [3]. The G*Power software (Version 3.1; Düsseldorf, Germany) was used for sample size calculations [42]. A questionnaire was used to determine the participants’ demographic information and their familiarity with basketball activity and/or any other related team sports. The inclusion criteria included: (i) registered in the secondary school supporting this study; and (ii) chronological age between 14–16 years. Exclusion criteria included: (i) playing basketball or any other team ball sports in a club (this criterion was adopted to prevent transferring effects across sports [43]); and (ii) current visual impairment. Participants were informed about the study’s scope, and their written informed consent to participate in this study obtained thereafter. This study was approved by the ethics committee of the Ministry of Education, Tunisia (approval code: 2173/2017). This study was undertaken in accordance with the Declaration of Helsinki and its later amendments in 2013.

### 2.2. Design

A 2 × 2 mixed design with factors “Condition” (video modeling vs. static pictures) and “Gender” (male vs. female) was used to investigate the hypotheses in this study. Participants were quasi-randomly (i.e., matched for gender) assigned to a dynamic condition (20 males, 20 females) and a static condition (20 males, 20 females).

### 2.3. Apparatus and Stimulus Information

The experiment was conducted using an HP Pavilion dv6 Entertainment PC placed at a distance of 30 cm from the participants. The stimuli (via PowerPoint software) were presented on a 32 × 20 cm screen, with a 45° viewing angle.

**Figure 1:**
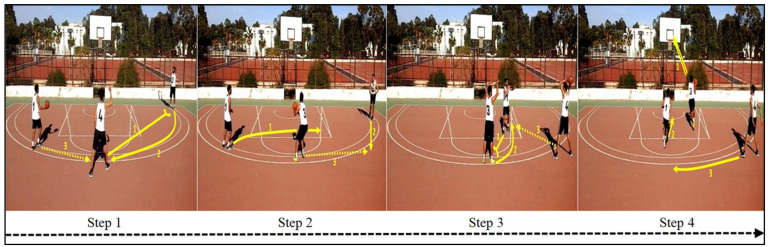
Sequence of four static pictures showing the four key steps of the basketball playing system.

Participants were requested to learn how to perform different tactical actions in basketball. A structured zone attack scene was developed in collaboration with two qualified teacher/basketball coaches (with over 12 years of experience). This playing system included three players (a playmaker ➀, a winger ➂, and a pivot ➃) who carried out a coherent tactical combination which was composed of three passes before a basket was taken through a layup. Each pass corresponded to a new step made up of multiple offensive actions achieved by the players (e.g., lateral movements, screening, and layup). Next, this game executed by three expert players (M_age_ = 21.7 years, SD = 1.26), serving as models, was filmed from a camera (using Samsung Galaxy Tab 3 SM-T211) placed above the ground from the middle of the field in an elevated position (approximately 2.5 m high). The recording position was set to film the entire field of play and all players’ actions. The recorded footage was transferred onto a computer via a fire-wire connection, and then was presented into a PowerPoint page. For the static presentation version, the continuous recording was divided into four static pictures, which depicted the key steps of the playing system. Photographs were captured using FastStone Capture 6.7 software (Barcelona, Spain), and play actions were denoted by the yellow numbered arrow-symbols. A dotted arrow refers to a simple pass; a solid arrow refers to a play movement; a double solid arrow refers to a layup; and a short perpendicular line at the end of a movement line refers to a screen. These static pictures enriched with arrows (820 × 972 pixels) were displayed simultaneously in one row into a PowerPoint page (see Figure 1). The dynamic and static presentations lasted for 12 s before vanishing from the screen, and they were system paced and purely visual (i.e., without any written/spoken commentary) in order to avoid a confounding occurrence of modality, redundancy, and temporal continuity effects [44].

### 2.4. Measurements

This study incorporated a control variable (i.e., spatial ability), and three dependent variables including, mental effort, game performance, and learning efficiency.

#### 2.4.1. Spatial Ability

**Figure 2:**
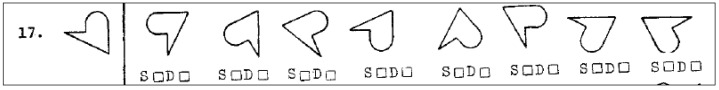
Example of a card rotations test item.

Students’ spatial ability was evaluated through the card rotations test (CRT) [45]. CRT is a true–false test including two parts of 10 questions; it was developed to evaluate an individual’s ability to see similarities and differences between the shapes. One point is given to each true answer, and the total scores could range from 0 to 160. Figure 2 shows one of the CRT items.

#### 2.4.2. Mental Effort

A 9-point scale ranging from (1) *very, very low mental effort* to (9) *very, very high mental effort*, was used to measure the mental effort invested during the study phase. This self-rating measure is valid and reliable for estimating cognitive load [46].

#### 2.4.3. Game Performance

A game performance task was performed in an outdoor basketball half-court, with two other male players (Mage = 16.22, SD = 1.2; semi-professional level with over 6 years of experience) who already knew the learning material. This test was conceived based on the recall–reconstruction paradigm [47]. Each tested student was instructed to reproduce as accurately as possible the tactical actions performed by a randomly chosen player from the learning material (i.e., playmaker, pivot, or winger). To guarantee the smooth running of the test, one of the semi-professional basketball players (used as teammates) was instructed to intervene by providing verbal corrective feedback each time the student performed a wrong action. A digital camera was used to record the students’ game performance. Then two independent raters (qualified teacher/basketball coaches) scored the total number of correct and incorrect positions/actions. One point was awarded for each correct position/action; otherwise, participants received 0 points (see Table 1). The scores could range from 0 to 8. The inter-rater reliability was excellent and satisfactory (Cohen’s κ = 0.91).

#### 2.4.4. Learning Efficiency

Learning efficiency was calculated based on Kalyuga and Sweller’s computational approach: Efficiency = Game performance/Mental effort [48]. These combined indicators have been seen as an optimal tool to evaluate learning from instructional visualizations [13], and have been used in previous studies assessing the effect of external visualizations on tactical learning in PE [3,4] and the sports coaching domain [49,50]. According to this computational approach, a lower mental effort investment combined with higher performance scores (and a same mental effort investment combined with higher performance scores; or vice versa) would provide evidence of a more efficient learning condition.

### 2.5. Procedure

**Figure 3:**
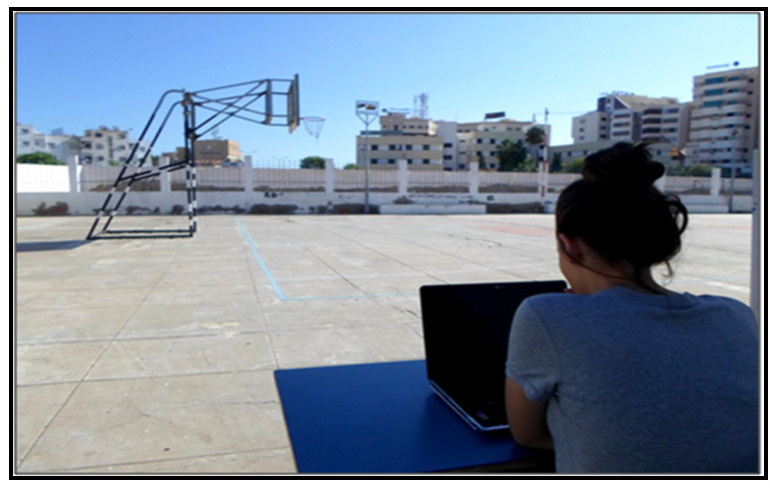
Learning phase.

The experiment was run in groups of 10 students in an outdoor basketball court. In each group, students were tested individually with the experimenter observing (±90 min), and no participant had the opportunity to observe the performance of another participant. First, each student was quasi-randomly assigned to one of the visual conditions and was instructed to memorize as precisely as possible the evolution of the scene of the play (*learning phase*). The scene of the play was shown twice resulting in a total duration of 24 s (Figure 3). Students exposed to the static pictures condition were initially informed of the functions of the arrows before watching the game situation. Immediately after watching either a static or dynamic visualization of the playing system, the student was given 30 s to indicate his/her mental effort investment level, 1 min to perform the game performance test, and 7 min to complete the card rotations test (*test phase*). The time was controlled by the experimenter using a handheld stopwatch.

### 2.6. Statistical Analyses

Statistical tests were processed using STATISTICA Software (StatSoft, Hamburg, Germany). Mean and SD (standard deviation) values were determined for each variable. After verifying that the assumptions required for parametric tests were not violated using the Shapiro–Wilk test for distribution normality, a two-way ANOVA [2 Conditions (video modeling vs. static pictures) × 2 genders (female vs. male)] with repeated measures was used to analyze the student’s spatial ability, mental effort investment, game performance, and learning efficiency. When ANOVA revealed a significant difference, a post-hoc Bonferroni was applied. The qualitative magnitudes were reported as partial eta squared ($n_{p}^{2})$ and Cohen’s mean standardized differences (*ES*) for post-hoc comparisons. The level of significance was set at *p* < 0.05. Following H’mida et al. [24], exact *p* values were reported, except when alpha level was >0.05 and/or <0.0005.

## 3. Results

Descriptive statistics for the control variable (i.e., spatial ability) for female and male students as a function of experimental conditions are presented in Table 2.

### 3.1. Spatial Ability

The results showed a non-significant effect of condition [*F* (1.19) = 0.07, *p* > 0.05, $n_{p}^{2}$ = 0.003], a significant effect of gender [*F* (1.19) = 47.89, *p* < 0.0005, $n_{p}^{2}$ = 0.99], and a significant interaction between these two factors [*F* (1.19) = 11.90, *p* = 0.0026, $n_{p}^{2}$ = 0.38]. Post-hoc analyses showed that the male students had significantly better spatial ability scores than the female students in the video modeling condition (*p* < 0.0005, *ES* = 12.69), and in the static pictures condition (*p* < 0.0005, *ES* = 11.77). Further analyses revealed no significant differences between the two conditions (video modeling/static pictures) for the female students (*p* > 0.05, *ES* = 0.60), and for the male students (*p* > 0.05, *ES* = 0.97). Consequently, the data analysis showed that spatial ability is higher in males than in female participants.

### 3.2. Game Performance

**Figure 4:**
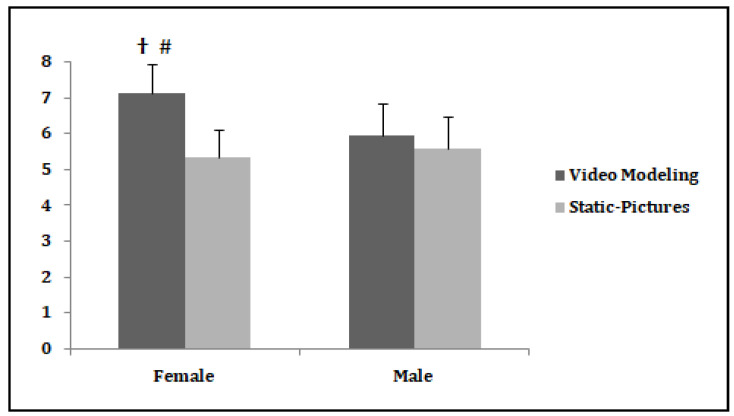
The game performance scores recorded for female and male students as a function of experimental conditions. ^#^ Significant difference between conditions for female students. ^†^ Significant difference between female and male students in the video modeling condition.

The game performance scores recorded for female and male students as a function of experimental conditions are presented in Figure 4.

The results showed a significant effect of condition [*F* (1.19) = 71.55, *p* < 0.0005, $n_{p}^{2}$ = 0.79], a significant effect of gender [*F* (1.19) = 14.94, *p* = 0.0010, $n_{p}^{2}$ = 0.44], and a significant interaction between these two factors [*F* (1.19) = 15.68, *p* = 0.0008, $n_{p}^{2}$ = 0.45]. Post-hoc analyses for the female students showed significant differences between the two conditions (*p* < 0.0005, *ES* = 2.30). It was found that females performed significantly better in the video modeling condition than in the static pictures condition. However, post-hoc analyses for the male students revealed no significant differences between the two conditions (*p* > 0.05, *ES* = 0.39). The males performed at the same level regardless of the instructional visualization (video modeling/static pictures) in which they were exposed. Further analyses showed that the female students had significantly better game performances than the male students in the video modeling condition (*p* = 0.0010, *ES* = 1.41). Otherwise, it was found that female and male students had similar game performances in the static pictures condition (*p* > 0.05, *ES* = 0.30).

### 3.3. Mental Effort

**Figure 5:**
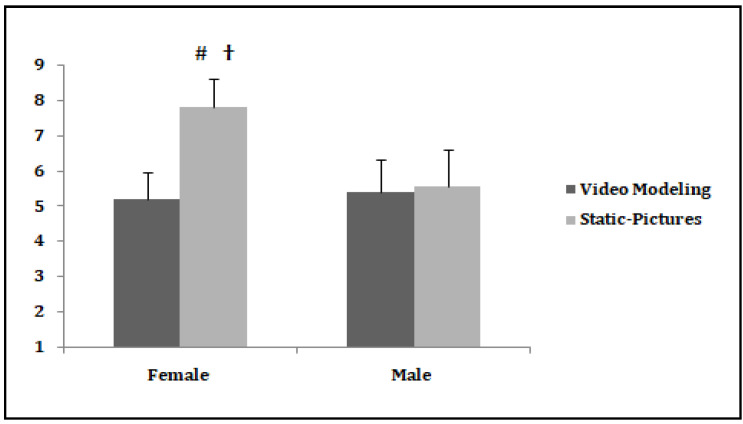
The mental effort scores recorded for female and male students as a function of experimental conditions. ^#^ Significant difference between conditions for female students. ^†^ Significant difference between female and male students in the static pictures condition.

The mental effort scores recorded for female and male students as a function of experimental conditions are presented in Figure 5.

The results showed a significant effect of condition [*F* (1.19) = 76.12, *p* < 0.0005, $n_{p}^{2}$ = 0.80], a significant effect of gender [*F* (1.19) = 32.62, *p* < 0.0005, $n_{p}^{2}$ = 0.63], and a significant interaction between these two factors [*F* (1.19) = 61.73, *p* < 0.0005, $n_{p}^{2}$ = 0.76]. Post-hoc analyses for the female students showed significant differences between the two conditions (*p* < 0.0005, *ES* = 3.25). It was found that females invested more mental effort in studying the static pictures condition than in the video modeling condition. However, post-hoc analyses for the male students revealed no significant differences between the two conditions (*p* > 0.05, *ES* = 0.15). The males invested the same amount of mental effort regardless of the instructional visualization (video modeling/static pictures) in which they were exposed. Further analyses showed that the female students had invested more mental effort than the male students in studying the static pictures condition (*p* < 0.0005, *ES* = 2.38). Otherwise, it was found that female and male students invested the same amount of mental effort in the video modeling condition (*p* > 0.05, *ES* = 0.23).

### 3.4. Learning Efficiency

**Figure 6:**
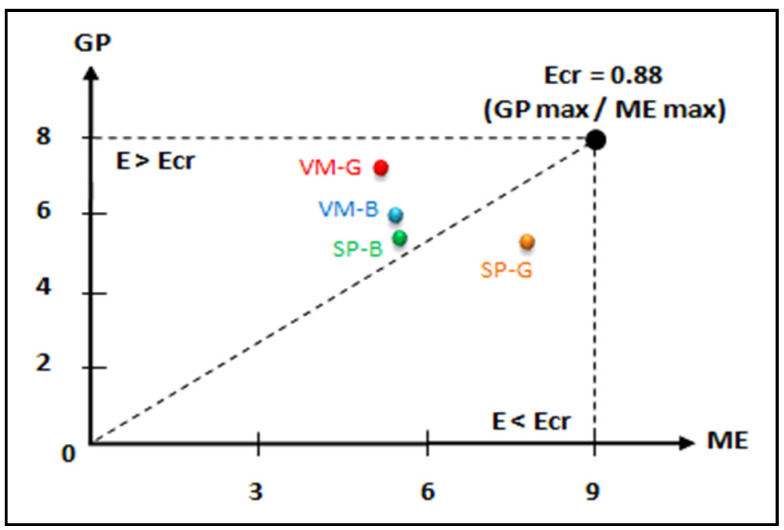
Learning efficiency per experimental condition for high-content complexity, based on mental effort and comprehension performance. The diagram is a representation modeled after Kalyuga and Sweller [48], where VM-G = video modeling girls, VM-B = video modeling boys, SP-G = static pictures girls, SP-B = static pictures boys, GP = game performance, ME = mental effort, E = efficiency, Ecr = critical efficiency.

The analysis showed a significant effect of condition [*F* (1.19) = 80.67, *p* < 0.0005, $n_{p}^{2}$ = 0.81], a non-significant effect of gender [*F* (1.19) = 0.67, *p* > 0.05, $n_{p}^{2}$ = 0.44], and a significant interaction between these two factors [*F* (1.19) = 38.99, *p* < 0.0005, $n_{p}^{2}$ = 0.67]. Post-hoc analyses for the female students showed significant differences between the two conditions (*p* < 0.0005, *ES* = 3.12). Therefore, for females, learning was more efficient in the video modeling condition than in the static pictures condition. However, post-hoc analyses for the male students revealed no significant differences between the two conditions (*p* > 0.05, *ES* = 0.36). The males achieved similar learning outcomes regardless of the instructional visualization (video modeling/static pictures) in which they were exposed. Further analyses showed that in the video modeling condition, learning was more efficient for the female students than for the male students (*p* = 0.0050, *ES* = 1.07). Otherwise, it was found that in the static pictures condition, learning was more efficient for the male students than for the female students (*p* = 0.0006, *ES* = 1.58). Representations based on Kalyuga and Sweller [34] of the learning efficiency measurement are illustrated in Figure 6.

## 4. Discussion

The study reported in this paper was designed mainly to explore how students’ gender could affect tactical learning in the PE domain, when processing an offensive basketball scene from video modeling and simultaneous static pictures. To examine the relationships between these two factors (i.e., visual representations and gender), a game performance test and a self-report of mental effort scale were used.

In line with the first hypothesis, the results showed that the video modeling does not lose its effectiveness in learning basketball tactical actions (whatever the gender of the learners), despite the addition of arrow-symbols which can allow learners to improve motor learning from static pictures [24,51]. These results are in line with a consistent body of research carried out either in sports or in other instructional domains, showing the positive effects of dynamic visualizations in learning when the content to be learnt is realistic and involves procedural motor knowledge [20,21,24,52]. Consequently, the human movement effect [27] has again been supported in learning sport skills requiring the whole body, indicating that video modeling examples were found to be within the working memory constraints of the students, and were not too long and/or complex to become subject to transient effects (see Wong et al. [14] for a discussion of how the transient nature of digital videos can sometimes hinder motor learning). As mentioned in the Introduction section, the instructional/cognitive benefits of dynamic visualizations (in comparison with statics) in learning/memorizing motor skills, were principally due to the activation of the mirror neuron system [25,26].

An additional crucial finding of the current study was the significant interaction between gender and visualization formats, indicating that while females performed better with the video modeling than with static pictures (i.e., they achieved higher game performances with less mental effort investment), males did not gain particular benefits from dynamic presentation (i.e., they achieved the same game performances with the same amount of mental effort investment). These results are consistent with previous research indicating that females profited particularly from dynamic visualizations (and males did not) in learning descriptive knowledge [38,39]. More importantly, our results are in accordance with Wong et al. [40] showing that females outperformed males with the video modeling, and that no gender differences were found with the static pictures, in learning a manipulative motor skill (i.e., Lego construction task). To explain these results, it is indispensable to refer to the student’s individual spatial ability that was evaluated as a control variable in our study through the CRT developed by Ekstrom et al. [45]. Indeed, it is interesting to note that males achieved better scores than females in the CRT, confirming the results of previous works indicating that spatial ability is higher in males than in females [33,34,35,36]. Consequently, our findings could support the “*ability-as-compensator hypothesis*”, indicating that low spatial ability learners (i.e., females) profited mainly from dynamic visualizations, and high spatial ability learners (i.e., males) did not [31,32,41]. Following this hypothesis, dynamic formats can further boost the learning of students with low spatial ability by offering an explicit and continuous representation of the spatio-temporal elements of the system, and thereby, avoiding the process of mental inference from static presentation formats. Yet, dynamic and static visualizations can have similar effects on learning among students with high spatial ability as they are more cognitively prepared to generate an adequate mental representation of the learning content whatever the presentation format [32]. Another plausible explanation for the superiority of female over male students in learning from video modeling could be related to the anatomical differences in the mirror neuron system. It is well known that the female brain has a higher proportion (compared to male brain) of gray matter in the prefrontal cortex [53,54], which play a crucial role in human attention [55]. As attention is the first necessary condition in any form of observing and modeling behavior [7], it is logical to have found that females achieved higher learning outcomes than males when processing a basketball playing system from video modeling examples. However, it should be cautioned that this study did not include any anatomical brain data, leaving it partially speculative. Further research is needed to explore this issue by using objective measures (e.g., [56]).

Some limitations should be mentioned as is the case for all experimental research. First, in the current study we focused solely on spatial ability as potential gender cognitive variables that can influence the learning from dynamic vs. static visualizations due to its large documented impact. However, it was established that there are both biological and social differences in the ways that males and females process external stimuli [57]. Second, the participants’ age may be a limiting factor for the generalization of our results’ interpretation. Additional research on this topic should perhaps explore this pattern of results with children or older participants (e.g., university PE students). Third, the current investigation was based on the conventional learning-and-recall experimental procedure [3]. In other words, participants were asked to perform the recall-performance test immediately after they had just watched one of the two visual supports. It would be worthwhile in future studies to examine findings of this study in a real-world setting (i.e., during regular PE lessons). Lastly, all participants undertook the experimental procedure at the same time of the day (i.e., in the morning). Further studies are required to investigate the effects of the time of the day and instructional visualizations (i.e., static vs. dynamic) in learning about basketball tactical skills, because it was established that cognitive abilities such as reaction time, attention, and executive functions depend heavily on the time of the day [58].

## 5. Conclusions

The current study extends the existing research examining the relationships between instructional visualizations and gender, indicating a significant interaction between these two factors in learning sport-specific motor skills (basketball tactical actions). For female secondary school students, learning was more efficient with video modeling than with static pictures. However, male secondary school students achieved similar learning performances with both types of instructional visualizations. In summary, this study suggests that a consideration of learners’ gender is crucial to further boost learning of basketball tactical actions from dynamic and static visualizations in adolescent students.

## Figures and Tables

**Table 1:** Different steps of the basketball playing system and their related success criteria.

Steps	Criteria	Scores	
		Action	Position
1	- The pivot ➃ at the level of 90° of the 3 point line on the central corridor makes a virtual pick to free the winger ➂ located at the level of 0° of the 3 point line on the right side corridor - The winger ➂ located at the level of 0° of the 3 point line on the right side corridor comes out on the pivot ➃ screen and moves at the level of 90° of the 3 point line on the central corridor - The playmaker ➀ located at the level of 45° of the 3 point line on the left side corridor passes the ball to the winger ➂	0 or 1 0 or 1 0 or 1	0 or 1 0 or 1 0 or 1
2	- The playmaker ➀ located at the level of 45° of the 3 point line on the left side corridor moves towards the “elbow” on the right side - The pivot ➃ located at the level of 0° of the 3 point line on the right side corridor moves at the level of 45° of the 3 point line on the right side corridor - The winger ➂ located at the level of 90° of the 3 point line on the central corridor passes the ball to the pivot ➃	0 or 1 0 or 1 0 or 1	0 or 1 0 or 1 0 or 1
3	- The playmaker ➀ located at the “elbow” on the right side makes a virtual pick to free the winger ➂ located at the level of 90° of the 3 point line on the central corridor - The winger ➂ located at the level of 90° of the 3 point line on the central corridor comes out on the playmaker ➀ screen and moves at the “elbow” on the right side - The pivot ➃ located at the level of 45° of the 3 point line on the right side corridor passes the ball to the winger ➂	0 or 1 0 or 1 0 or 1	0 or 1 0 or 1 0 or 1
4	- The winger ➂ located at the “elbow” on the right side perform a layup - The playmaker ➀ located at the level of 90° of the 3 point line on the central corridor moves for a rebound - The pivot ➃ located at the level of 45° of the 3 point line on the right side corridor moves at the level of 90° of the 3 point line on the central corridor	0 or 1 0 or 1 0 or 1	0 or 1 0 or 1 0 or 1

**Table 2:** Means and (standard deviations) for spatial ability, as a function of condition and gender.

	Video Modeling	Static Pictures
Male	139.4 (2.98) ^†^	142.8 (3.97) ^†^
Female	92.3 (4.32)	89.45 (5.03)

† Significant difference between male and female students in the same condition.

## Data Availability

The data are available upon request to the corresponding author’s email.
